# Dissecting Host-Pathogen Interactions in TB Using Systems-Based Omic Approaches

**DOI:** 10.3389/fimmu.2021.762315

**Published:** 2021-11-02

**Authors:** Khushboo Borah, Ye Xu, Johnjoe McFadden

**Affiliations:** School of Biosciences and Medicine, Faculty of Health and Medical Sciences, University of Surrey, Guildford, United Kingdom

**Keywords:** *Mycobacterium tuberculosis*, tuberculosis, systems biology, macrophage, omic technology

## Abstract

Tuberculosis (TB) is a devastating infectious disease that kills over a million people every year. There is an increasing burden of multi drug resistance (MDR) and extensively drug resistance (XDR) TB. New and improved therapies are urgently needed to overcome the limitations of current treatment. The causative agent, *Mycobacterium tuberculosis* (Mtb) is one of the most successful pathogens that can manipulate host cell environment for adaptation, evading immune defences, virulence, and pathogenesis of TB infection. Host-pathogen interaction is important to establish infection and it involves a complex set of processes. Metabolic cross talk between the host and pathogen is a facet of TB infection and has been an important topic of research where there is growing interest in developing therapies and drugs that target these interactions and metabolism of the pathogen in the host. Mtb scavenges multiple nutrient sources from the host and has adapted its metabolism to survive in the intracellular niche. Advancements in systems-based omic technologies have been successful to unravel host-pathogen interactions in TB. In this review we discuss the application and usefulness of omics in TB research that provides promising interventions for developing anti-TB therapies.

## Introduction

Tuberculosis (TB) remains a global pandemic and the biggest infectious killer despite being a preventable and curable disease (1). This communicable disease is caused by the pathogen *Mycobacterium tuberculosis* (Mtb) and it kills over a million people every year. In 2016, WHO estimated around 1.7 billion people were reported to have latent TB infection (1, 2). Drug resistance in TB has been an escalating problem worldwide with nearly half a million people developing rifampicin resistance in 2019. The pathogenic mycobacterium becomes resistant to the first line anti-TB drugs including isoniazid, rifampicin, fluoroquinolone and second line drugs causing multidrug resistant (MDR) and extensively drug resistant (XDR) TB, which are a serious global threat with MDR-TB accounting for one third of the deaths worldwide due to antimicrobial resistance (1, 3). MDR and XDR-TB cases are developed mainly due to poor drug regimen, treatment misuse and poor patient compliance due to the lengthy anti-TB treatments and the associated side-effects of the medications (3). This rapidly growing problem of drug resistance needs urgent attention. We need to develop new therapies to overcome the limitations of current anti-TB drug treatments. We also need improved and efficient diagnostic platforms for detection and surveillance for disease control and management of MDR and XDR-TB. COVID-19 pandemic posed significant challenges and disruptions to TB control and cure by restricting the health services, infrastructure, workforce and research efforts which were diverted away from TB and other diseases (4, 5). These disruptions from COVID-19 pandemic are predicted to increase global TB deaths by 20% over the next 5 years (4). We need to harness global research efforts to develop new and effective treatments for TB and to mitigate the limitations on treatment and cure imposed by the COVID-19 pandemic for avoiding additional morbidity and mortality.

**Figure 1:**
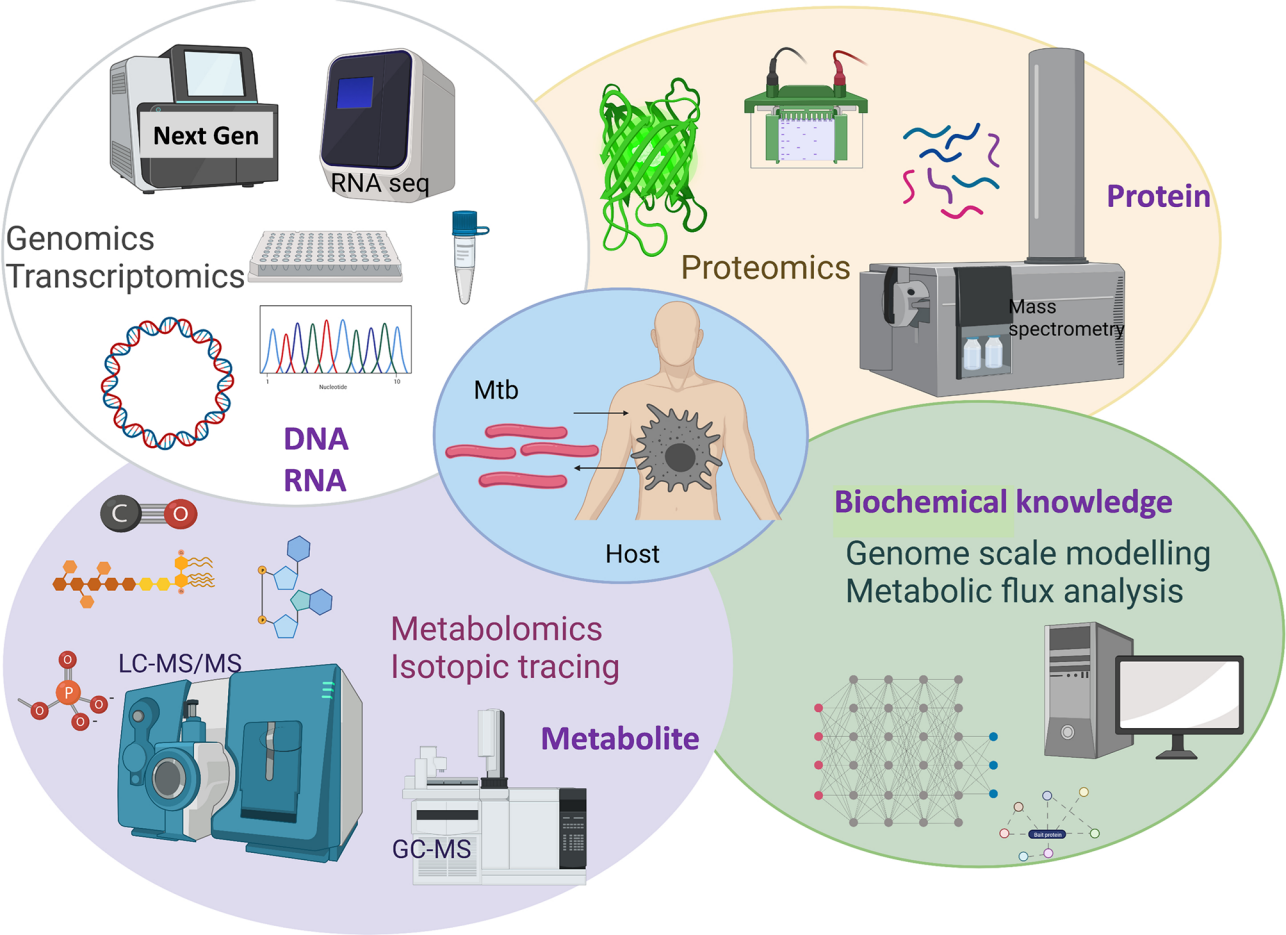
Overview of systems-based omics used for exploring Mtb and host metabolic interactions. Created with BioRender.com.

The causative agent of TB has evolved to persist in humans. Mtb interacts with alveolar macrophages which are the primary cellular site of infection (6, 7). Macrophages in the lungs of an infected individual phagocytize TB bacilli which were inhaled as aerosols from another infected individual (6). Inside macrophages Mtb are contained in phagosomes which exhibit bactericidal activity through maturation and acidification (8, 9). Macrophages produce reactive oxygen and nitrogen species to eliminate intraphagosomal Mtb (6, 10). Macrophages initiate a pro-inflammatory response with other immune cells being recruited to the site of infection and forming a granulomatous lesion to arrest bacterial replication and stop dissemination. However, Mtb has evolved adaptation mechanisms to avoid the hostile host environment and immune defences including intraphagosomal survival and escape into the cytoplasm (9, 11). Mtb adapts its life and metabolism to survive in the hostile phagosome environments including hypoxia, low pH, antimicrobial peptides, reactive oxygen and nitrogen species and nutrient limitations (12–14). Metabolism of the TB pathogen is key for its survival in the human host. Decades of research using *in vivo* and *in vitro* TB models has enabled identification of the metabolic characteristics of Mtb within the intracellular niche. It is now well-established that Mtb uses multiple host metabolites such as lipids and amino acids as nutrient sources during infection (6, 13–15). Therefore, identifying vulnerable metabolic targets of Mtb provide avenues for the development of new antimicrobials and it has been an accelerating research area. Scientific advances in the development and application of systems-wide tools and platforms to screen genome, transcriptome, proteome, metabolome, metabolic modelling and fluxome have revolutionised research in untangling the interactions between human host and Mtb (**Figure 1**). In this review, we discuss advancements in TB research achieved using systems-based omics.

## Genomics, Transcriptomics, and Proteomics

The discovery of next-generation sequencing (NGS) facilitates the genomic and transcriptomic research of mycobacteria. The genomic or transcriptomic analysis focuses on the variation of genomic features, such as gene expression level, DNA sequencing or regulatory elements annotation, which enables the identification of essential genetic or regulatory targets under certain conditions (16–18). Resistance mutation of mycobacterium to drugs can be detected by genomic analysis of clinical isolates (19). The massive online database generated from genomics analysis of resistance mutation enables the generation of genetic interaction networks to different antibiotics (18, 20). RNAseq and methylome analysis of different clades of Mtb complex detected differential gene expression involved in host interaction and metabolism, which is further linked to the varied phenotypes and host susceptibility between the Mtb complex (21). RNAseq has been used to identify the relevant virulent genes in clinical Mtb strains such as the MKR mutant (17). Genes involved in cholesterol degradation as well as ESX-1 secretion system were up-regulated in the MDK strain but not in Mtb H37Rv (17). Drugs targeting the cellular pathway could decrease the intracellular survival of the MDK strain, which further proves the application of transcriptional analysis of Mtb strains could facilitate the identification of drug targets. Some studies combining both genomics and transcriptomic analysis among the same set of mycobacterial isolates identify specific single nucleotide polymorphisms (SNPs) and varied gene expression levels, as well as the link to phenotypes of the bacterial population (22, 23). Genomics or transcriptomics analyses are adopted to investigate the influence of deletion or over-expression mutants on the gene expression of the whole population to identify relevant cellular pathways (24, 25). Genomics and transcriptomics have provided insights into TB pathogenesis and host-pathogen interactions and facilitated identification of biomarkers of TB and vaccine development (26–29). RNA seq analyses by Kaufmann et al., demonstrated changes in the transcriptome profiles of hematopoietic stem cells (HSCs) and multipotent progenitors in BCG intravenously vaccinated mice *vs*. control mice. These epigenetic changes in the HSCs enhanced myelopoiesis and generated BCG trained macrophages that provided enhanced protection against Mtb (27). Esterhuyse et al. employed DNA methylome, transcriptome and proteome analyses of monocytes and granulocytes to demonstrate differences in DNA methylation profiles which correlated to changes in immune cell activation, transcription and inflammation between active TB and latent TB infection (LTBI) patients (29). A recent study using transcriptomic analyses compared the lung biosignatures between TB, lung adenocarcinoma (LUAD) and sarcoidosis patients and identified *MK167* which is a mediator of Mtb-promoted tumor cell proliferation, migration, and invasion to be overexpressed in both TB and LUAD patients (26).

Proteomic analyses are used to identify and quantify proteins, which are functional biochemical entity of an organism, and these analyses provide direct functional information complementary to the genomics and transcriptomics analyses (30). Development and application of proteomic platforms has been researched for over a decade. In TB research, systems-level proteomic analysis has been applied with the motive to develop diagnostic markers, vaccines and therapeutics (31). Proteomic methods involve gel electrophoresis separation of proteins (30, 32) and liquid chromatography-mass spectrometry (LC-MS/MS) analyses of protein samples which can be conducted, broadly in two ways. A widely used approach, also known as bottom-up proteomics, involves digestion of proteins into peptides, followed by their identification and mapping into the proteins using their mass-to-charge ratios and further fragmentation of the ions for quantitative analyses (30, 33). The second approach, called top-down approach involves fragmentation of the total intact protein into molecular ions followed by protein identification and quantitation (31). Proteomic studies in TB have provided information on the TB pathogen’s protein expression profiles in *in vitro* and within the host. Discovery proteomic analyses by Rosenkrands et al. (34) identified 82 novel proteins out of 49 extracellular culture filtrate and 118 cellular lysate proteins in Mtb. Comparative proteomic analyses in clinical isolates and virulent and avirulent mycobacterial vaccine strains and Mtb complexes (MTBC) have revealed strain-specific characteristics (35, 36). Jungblut et al. (35) identified six proteins including L-alanine dehydrogenase (Rv2780), isopropyl malate synthase (Rv3710), nicotinate-nucleotide pyrophosphatase (Rv1596), MPT64 (Rv1980c) and two conserved hypotheticals (Rv2449c and Rv0036c) with missing counterparts in *M. bovis* BCG (Chicago strain). Chicago and Copenhagen BCG strains exhibited highly similar proteomes with only three identified variants while Mtb H37Rv and Erdman strains had 18 variants between them (35). Clinical strains JAL and BND showed distinct variations in the *Esx* and *mce1* operon proteins, which contributed to their virulence and drug resistance, as compared with H37Ra and H37Rv strains (37). A tandem mass tag (TMT) labelled proteomics identified differential protein expression and phosphorylation in PE/PPE/PE-PGRS between H37Rv and H37Ra which contributes to the virulence of H37Ra in human cells (38). Differences in expression of nitrate metabolism proteins between Mtb H37Rv and drug-susceptible Beijing and multidrug-resistant Beijing strains were identified to be important in the pathogens’ adaptation to stressful intracellular environments (39). The non-replicative persistent (NRP) state of Mtb was investigated using isotope coded affinity tag-based (ICAT) proteomics which uses isotopic labels for quantitation of proteins (40). Cho et al. (40) cultivated Mtb in an oxygen depleted fermentor to achieve early and late NRP states of cultures and measured relative expression of proteins. This proteomic analysis revealed different expression profiles in the two NRP states which were associated with energy metabolism and degradation (40). In guinea pig models of TB, shot gun tandem mass spectrometry (MS/MS) proteomics at early (30 days-post infection) and chronic (90 days-post infection) infection stages identified over 500 Mtb proteins in infected lung tissues (41). This research identified heterogeneity in two protein classes, belonging to the cell wall processes and respiration and metabolism between the two stages highlighting these processes as necessary adaptations in persistent Mtb (41).

Proteomic approaches have been used to investigate host-pathogen interactions and immunological responses in TB, and in identification of diagnostic markers. The cell wall of Mtb is important for its virulence the cell wall lipids and proteins are antigenic and elicit host immunomodulations. Lipoproteins, T and B cell antigens and proteins associated with small and macromolecule metabolism were identified in Mtb’s cell wall which likely facilitated the transport of nutrients between the cytosol and extracellular milieu and cell wall re-modelling (42). Antibody screening and immunological responses using systems level proteomics has attracted great attention in recent years. TMT based quantitative proteomics showed differentially expressed proteins in THP-1 macrophages infected with Mtb H37Rv and H37Ra strains, and include proteins involved in apoptosis, blood coagulation and oxidative phosphorylation providing evidences to strain-specific host macrophage responses (43). T-cell IFN- Ɣ immunological response elicited by Mtb antigens in culture filtrate and cellular extracts were screened in splenocytes from Mtb-infected mice which allowed identification of 17 novel T-cell antigens (44). Proteomic analyses by Penn et al. using affinity tag purification mass spectrometry mapped 187 Mtb-human protein-protein interactions and identified two factors, Mtb’s secreted protein LpqN and the human ubiquitin ligase CBL involved in Mtb’s pathogenesis (45). Robust quantification of proteins in complex samples representing the intracellular milieu is critical to not only advance the TB biology but also to develop therapeutic and diagnostic interventions. Schubert et al. (46) developed Mtb proteome library, a public resource of definitive MS assays using single reaction monitoring (SRM) technique to quantify proteins in complex biological samples. Label-free quantitation of plasma proteins in patients with pulmonary (PTB) and (LTBI) provided a diagnostic model which showed alpha-1-antichymotrypsin(ACT), alpha-1-acid glycoprotein 1 (AGP1), and E-cadherin (CDH1) with >80% sensitivity, specificity and accuracy in distinguishing LTBI from PTB (47). Another study using TMT-based quantitative proteomics of human serum demonstrated different signatures of inflammatory proteins and apolipoprotein A and serotransferrin proteins (involved in lipid transport and iron metabolism) in LTBI and active TB cohorts (48). A multidimensional and stable isotope labelled q3D LC-MS quantitative plasma proteomics enabled discovery of novel protein biomarkers and 5-protein signature comprising of complement factor H related 5 (CFHR5), interleukin enhancer binding factor 2 (ILF2), leucine-rich alpha-2 glycoprotein (LRG1), LPS-binding protein (LBP), serum amyloid A (SAA), plasma C-reactive protein (CRP) and E3 ubiquitin-protein ligase listerin (LTN) to improve diagnostic accuracy of TB (49). Immunological proteome screening using integrative host and pathogen biochemical datasets allowed identification of antibodies in human serum of TB patients. A large-scale screening used protein microarray platforms which included 4099 Mtb proteins; these microarrays were probed with serum from over 500 individuals with and without active TB. Antibodies were screened against the entire Mtb proteome to identify immunoproteome for active TB which were extracellular proteins comprising 0.5% of the entire proteome (50) demonstrating well-established application of proteomics in identification of host-pathogen interactions for serodiagnostic markers.

## Metabolomics and Isotopic Tracing Studies

Metabolomics is the one of the newest omic technologies used to discover, identify, and quantify metabolites which are biochemical entities produced and consumed in metabolic reactions which in turn drives metabolism and energy production in biological systems. Metabolomics allow big data integration across different omics for comprehensive determination of the consequences of all metabolites on cellular function and physiology (51–53). Metabolomics combined with isotope labelling strategies have identified nutrient sources for Mtb in *in vivo* environments and measured their uptake and assimilation by Mtb metabolic network; this is not feasible with any other omic platforms. Metabolomic analysis involves quenching of metabolites from living cells and cellular extracts and polar/nonpolar metabolites recovery, followed by analytical identification and quantification. Gas chromatography-mass spectrometry (GC-MS), nuclear magnetic resonance (NMR) and LC-MS are three most widely used analytical platforms for metabolomics. Depending on the purpose of the study, metabolites are analysed either by untargeted or unbiased methods/platforms, mainly used for qualitative analysis or by targeted analyses involving SRM or multiple reaction monitoring (MRM). Metabolism is at the heart of survival, growth, and virulence of Mtb, and other pathogens inside the human host cells. Metabolism of immune cells is equally important in driving a variety of cellular and immunological responses during infection and therefore, in recent years, immunometabolism has emerged as a rapidly growing area with the motive to develop new diagnostics and therapeutics for TB. The advancements in analytical tools and platforms have also allowed metabolomics and metabolic investigations at single cell and subcellular (cellular microcompartment) levels (54, 55). In this section, we have reviewed the latest developments in metabolomic technologies and their application to achieving breakthroughs in characterisation of the metabolic cross talk between the host and Mtb.

Metabolomics has been extensively used to identify metabolic pathways and to decipher metabolism of Mtb in *in vitro* and *in vivo* animal models. Activity-based metabolomic profiling which uses cellular metabolome as the platform using protein and time-dependent production and consumption of small molecules, assigned functional and structural characteristics of Rv1692 as a glycerol 3-phosphate phosphatase in glycerophospholipid metabolism (56). Rv322c has been recently assigned a nitrogen metabolic role in aspartate biosynthesis and this enzyme is essential for Mtb’s survival in murine macrophages and in mice (57). Lineage-specific metabolic differences, predominantly in amino acid, glycolysis, and tricarboxylic acid (TCA) cycle metabolism between six strains of MTBC complex were identified in exometabolomes of culture supernatants by untargeted time-of-flight (TOF) MS analysis. This study involved integration of metabolomics data into a constraint-based model which predicted the SNPs and genetic basis of the metabolic variations (58).

Complementary to other omic platforms, metabolomics has been applied to successfully identify Mtb and host metabolites in human biological fluids. High resolution orbitrap MS identified lipid metabolites such as phospha-tidylglycerol (16:0_18:1), lysophosphatidylinositol (18:0) and acylphosphati-dylinositolmannoside (Ac1PIM1) elevated in plasma of patients with active pulmonary TB (59). Another study applied orbitrap MS/MS and measured host responses to active TB and LTBI and identified elevated tryptophan catabolism to kynurenine mediated by indoleamine 2,3-dioxygenase-1 (IDO-1) (60). Metabolic biosignatures measured in human urine samples identified pre and post therapy clinical responses to TB antimicrobials which included 23 molecular features changing during the treatment (61). Drug phenotypes and their mechanism of Mtb's growth inhibition has been investigated using metabolomics and isotopic labelling analyses. Bedaquiline (BDQ), a newly approved anti-TB drug exhibits Mtb killing through inhibition of ATP synthase. The mode-of-action of BDQ is complex and has been investigated by multiple research demonstrating that BDQ induces Mtb’s glycolytic and gluconeogenic metabolic dependencies and that pyruvate phosphate dikinase (PPDK) is a vulnerable metabolic target (62). Knoll et al. (63) measured metabolite markers involved in TCA cycle metabolism, cell wall and DNA synthesis with GCxGC-TOF-MS in Mtb grown with and without ciprofloxacin, (a quinolone potent against drug resistance TB) to identify mechanism of action and Mtb’s adaptation to antimicrobials (63). Comparison of drug resistant and drug susceptible metabolic profiles showed proline and isoleucine levels significantly reduced in drug resistant strains (64).

Host and pathogen interaction in infectious diseases has been investigated in detail by multiple studies across decades using metabolomics combined with isotopic tracing studies. Metabolite profiling and ^13^C tracer studies have re-assigned identity and functionality of Mtb enzymes and elaborated its central carbon metabolic (CCM) pathways such as Rv1248c as 2-hydroxy-3-oxoadipate synthase catalysing C-C bond formation between α-ketoglutarate and glyoxylate (65), the role of phosphoenolpyruvate carboxykinase (PEPCK) in gluconeogenesis and its essentiality for Mtb’s survival in mice models (66) and the bifunctional role of Rv0812 coupling nucleic acid and cell wall biosynthesis (67). Untargeted metabolite profiling and isotopic analysis of Mtb grown on ^13^C-labelled carbon substrates dextrose, acetate and glycerol demonstrated that Mtb catabolizes multiple carbon substrates simultaneously to achieve monophasic growth providing a snapshot of metabolic adaptations in Mtb to host intracellular niche (68). GC-MS based isotopologue profiling using ^13^C carbon isotopic labels of Mtb infected-human THP-1 macrophages measured utilization of glucose by Mtb and essentiality of the anaplerotic node enzymes pyruvate carboxylase (PCA), PEPCK, malic enzyme (MEZ), and PPDK in gluconeogenesis, propionate and cholesterol detoxification and lipid synthesis (15, 69). Similar isotopologue analyses have been successfully applied across various host-pathogen models to measure intracellular metabolism of these pathogens, such as ^13^C glucose profiles of *M. leprae* growing in Schwann cells (70) and *Trypanosoma brucei* in bloodstream form (71) and ^13^C glycerol profiles in *legionella pneumophila* replicating in macrophages (72). ^13^C-isotopic analysis of Mtb *in vitro* cultures in a chemostat setup at controlled growth rate demonstrated efficient isotope incorporation and co-catabolism of ^13^C-labelled acetate and cholesterol, which are intracellular nutrient sources for Mtb (73). Recent advances in MS technologies have enabled the use of several stable isotopes including both single and mixed labelled species such as ^15^N and ^2^H. ^2^H cholesterol labelling of Mtb *in vitro* cultures confirmed that Mtb utilized cholesterol to synthesize amino acids (73). ^15^N-asparagine was used to measure the uptake and assimilation of nitrogen in murine macrophages using NANO-Secondary ion mass spectrometry (SIMS) (74). Agapova et al. (75) performed an LC-MS and ^15^N-isotopologue analysis to measure ammonium and amino acid utilization in *in vitro* Mtb and demonstrated that amino acids were preferred over ammonium and were utilized at similar rates, and that alanine dehydrogenase *ald* was important for alanine utilisation as a nitrogen source (75). ^15^N-isotopic labelling and GC-MS isotopologue analyses of Mtb infected-THP-1 macrophages identified nitrogen assimilation of amino acids in intracellular Mtb replicating human cells and further identified phosphoserine transaminase *serC* as a potential drug target (13).

## Constraint-Based Modelling

Metabolism in a biological system involves numerous complex biochemical processes. Each biochemical reaction involves consumption and production of metabolites or chemical species. Metabolic flux through a reaction defines the rate at which a substrate is utilized and is the accurate measure of the activity of a pathway in the network. Metabolomics provides a measure of the pool sizes of the metabolites that can indicate active pathways but cannot measure the fluxes through the pathways. Therefore, measuring metabolic fluxes is the way to confirm network activity and function, and define the metabolic phenotype of a system.

Mathematical modelling of Mtb is a recent innovation in TB research and is popular for prediction of genes, metabolic and drug phenotypes of the pathogen and host-pathogen interactions. These models include a network of biochemical reactions including enzymatic, spontaneous or transport reactions which are reconstructed from annotated genome and literatures and implements computational methods such as flux balance analysis (FBA), flux variability analysis (FVA) and gene essentially predictions (76). Constraint based *in silico* modelling comprises of stoichiometric, mass, charge and energy balanced reactions and is used for prediction of steady-state phenotypes. The models include an objective function, typically a biomass equation. Flux distributions through the network are computationally optimized to maximize biomass function and growth rate to predict phenotypes. The accuracy of these predictions needs to be further validated using experimental approaches. These genome scale models are powerful as these platforms can be used to integrate transcriptomic, proteomic, and metabolic data to provide a holistic view of an organism’s metabolic and biochemical network in various environmental and nutritional conditions, stress and disease which is otherwise very challenging to derive from experimental and previously discussed omic platforms alone. Such metabolic modelling has been applied to study metabolic flexibility of Mtb and to predict mutant phenotypes (76–78). GSMN-TB, the first genome scale model of Mtb predicted the requirement of the enzyme isocitrate lyase during slow growth of Mtb in a continuous culture (78). Bonde et al. developed a differential producibility analysis (DPA) algorithm to extract metabolite production profiles through integration of micro array gene expression data into GSMN-TB model (79). DPA using GSMN-ML, the first genome scale model of *M. leprae*, was applied to interrogate RNA-seq data to derive *in vivo* metabolite production of leprae bacillus and nutritional status (80). Metabolic variations within the Mtb complex were identified through comparisons of substrate utilisation rates, gene essentiality data and growth rates calculated from GSMN-TB, GSMN-MB (*M. bovis*) and GSMN-BCG (*M. bovis* BCG) models (81). Lofthouse et al. tested a range of carbon and nitrogen sources to compare substrate utilisation between the models; the experimental validations identified several discrepancies with the predictions, highlighting the need to verify *in silico* predictions and to continually update and curate the models with updated genetic and enzymatic knowledge. Recent integration of metabolomic and genomic data into the genome scale models of Mtb complex predicted genetic mutations and their associated metabolic vulnerabilities in 18 clinical strains and providing an alternative approach to the genomic approaches such as GWAS to relate genetic variations to clinical phenotypes, and additionally, predicting related metabolic variations (53). Genome scale modelling has been used to identify drug targets through gene essentiality predictions (77). Rienksma et al. modelled metabolic state of Mtb during infection using condition-specific objective function and predicted nutrient uptake and gene essentiality to identify vulnerable drug targets in Mtb (76, 77). Till date, there are 16 genome scale models that vary in network topology and biomass equations, and these inconsistencies amongst the existing models poses limitations in their use and applications. It is therefore important that these models are updated and are standardized for predicting physiologically relevant phenotypes. Kavvas et al. (82) unified sMtb and iOSDD, two of the newest reconstructions and generated iEK1011, a standardized model that explored metabolic phenotypes of Mtb under various environmental conditions (82). Recently López-Agudelo et al. (83) conducted useful comparisons between eight Mtb models and identified the two best sMtb and iEK1011 models for metabolic state predictions (83). Although constraint-based genome scale modelling provides useful predictions on metabolic network operation and gene essentialities, these predictions are calculated based on the maximization or minimization of the assumed objective function in the model and therefore may not be an exact representation of the metabolic phenotype (84). The predictions need to be validated through measurement of metabolically active fluxes supported by experimental data.

## Metabolic Flux Analysis and Fluxomics

**Figure 2:**
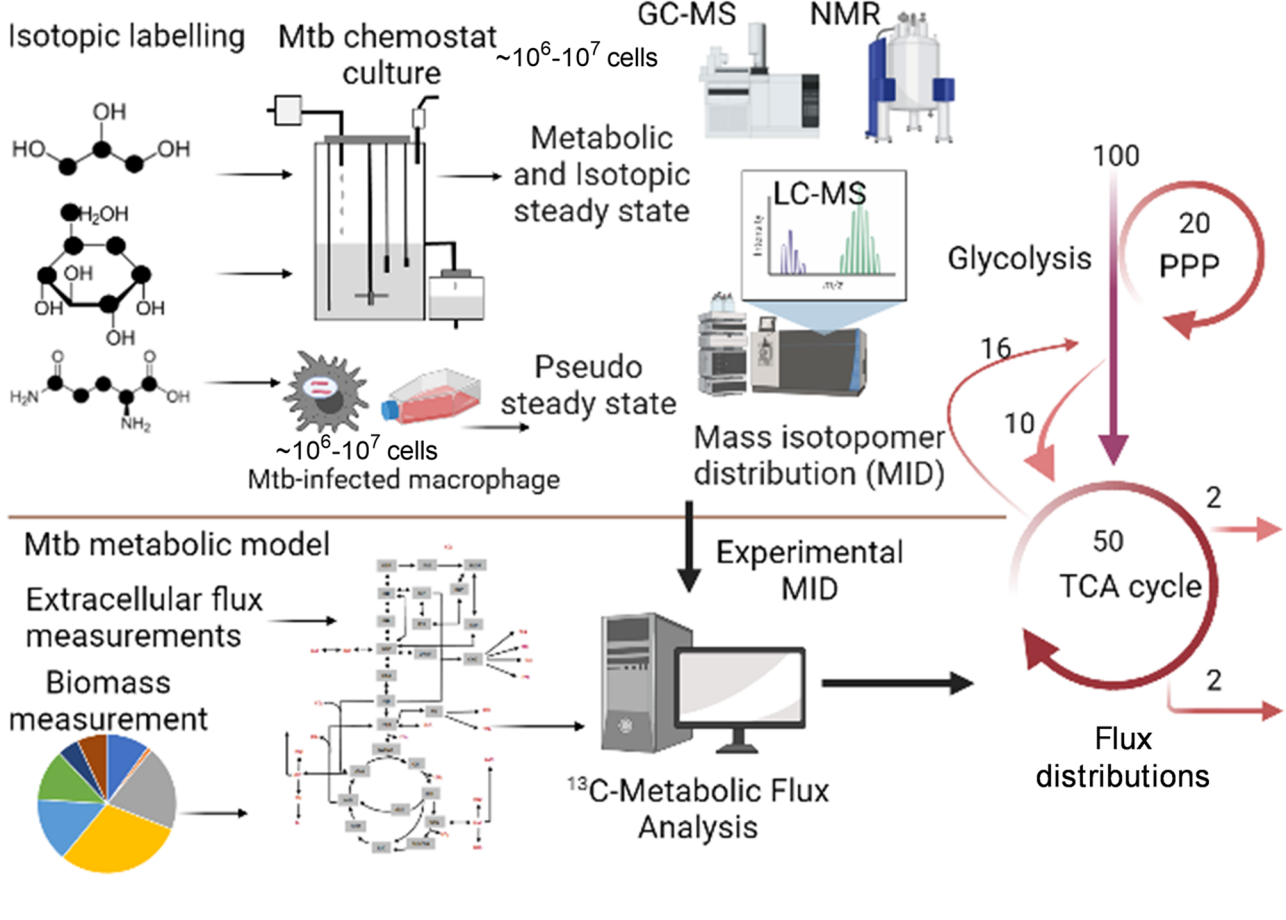
General workflow of steady state ^13^C-Metabolic Flux Analysis (MFA). The application is shown for measuring metabolic fluxes of Mtb *in vitro* in chemostat and during intracellular growth in macrophages. The methodology includes growth of the biological system in isotopically labelled media, followed by achievement of metabolic and isotopic steady state for chemostat set up or assuming pseudo steady state from macromolecular measurements at various time points of isotopic labelling. Metabolomics and mass isotopomer distributions (MIDs) are measured using GC-MS, LC-MS or NMR. A minimum of 10^6^-10^7^ cells is required for robust MID measurements. The computational part of ^13^C-MFA includes construction of a metabolic model consisting of atomic transitions for central metabolic reactions. The model is constrained with extracellular flux and biomass measurements. Measured MIDs are incorporated into the model and the model is cimulated with MFA computational platform to derive best-fit metabolic fluxes that defines the metabolic phenotype of the system under study. Created with BioRender.com.

Metabolic Flux Analysis (MFA), which is an amalgamation of computational and experimental techniques, provides precise quantification of metabolic fluxes in the network. The general workflow of steady state MFA is elaborated in **Figure 2**. The experimental part of MFA involves cultivation of microbial or eukaryotic cells in a growth medium containing isotopically labelled substrates. Majority of the MFA studies are conducted on steady state cultures where the cells are grown at a controlled growth rate using a bioreactor/chemostat setup (73, 85, 86) or assuming a pseudo-steady state during exponential growth mainly for eukaryotic cells (87, 88). Metabolic steady state of the system is confirmed through measurement of substrate uptake, CO_2,_ and biomass production rates (73). Metabolically steady state cultures are next checked for isotopic steady state through addition of isotopic substrates and their incorporation into the biomass at various time points of cultivation. Cells at isotopic steady state are harvested for extraction of metabolites and follow up metabolomic and mass isotopomer distribution (MID) analyses using mass spectrometry (GC-MS, LC-MS) or NMR. The computational part of MFA involves construction of a metabolic model of the system under study; the model includes atomic transitions for the central metabolic network reactions, free and measured fluxes and a biomass reaction constructed from experimentally derived measurements of macromolecular compositions required to constrain the model. The MIDs are included in the model as the experimental data set, and the model is simulated using linear programming to iteratively fit the experimental MIDs and derive intracellular fluxes. Several computational tools such as 13C-FLUX2 (86), Isotopomer Network Compartmental Analysis (INCA) (73), WUFlux (89) are available for MFA calculations and for statistical evaluations of measured fluxes.

Steady state ^13^C-MFA, which uses ^13^C-isotopically labelled substrates has been used to quantify intracellular central carbon metabolic fluxes of Mtb *in vitro* and in human THP-1 macrophages. ^13^C-MFA of Mtb *in vitro* cultures cultivated at slow and fast growth rate using ^13^C-glycerol as the labelled carbon substrate identified a novel pathway for pyruvate dissimilation through glyoxylate shunt and anaplerotic reactions and demonstrated the ability of Mtb to fix CO_2_ for synthesizing biomass (86). Recently, the intracellular carbon fluxes that support co-catabolism of multiple carbon substrates by Mtb were identified using ^13^C-MFA (73). This study demonstrated that flux partitioning between the TCA cycle and glyoxylate shunt with a reversible methyl citrate cycle enables Mtb to co-catabolise cholesterol and acetate. ^13^C-MFA derived metabolic flux profiles of BDQ-treated Mtb identified the operation of a bifurcated TCA cycle and requirement of the anaplerotic node and methylcitrate cycle, thereby elaborating the metabolic changes in BDQ-treated Mtb (62). Application of classical ^13^C-MFA to measure metabolic fluxes of Mtb inside human cells is challenging as the system is at non-steady state. To overcome this challenge Beste et al. (15) developed ^13^C-Flux Spectral Analysis (FSA), a computational tool that probed the uptake and utilisation of carbon sources including a mixture of amino acids, C1 and C2 compounds by Mtb from human THP-1 macrophages (15). In addition to carbon, Mtb utilises several nitrogen sources during infection. This was recently demonstrated by Borah et al. (13) through the application of ^15^N-isotopically labelled amino acids to Mtb-THP-1 macrophage and development of ^15^N-Flux Spectral Ratio Analysis (FSRA) to identify the nitrogen sources acquired by Mtb from the host cell and their intracellular assimilation (13). This research demonstrated glutamine as the primary nitrogen donor for intracellular Mtb and that serine biosynthesis is essential for survival of Mtb in human macrophages.

## Future Perspectives and Conclusions

Till date, several omic technologies have been developed and applied to investigate host-pathogen interactions in TB and in other diseases. Each of the five omics discussed in this review uses unique tools and methodology and specializes in the measurement of particular type of biomolecules and their functions. Genomic technologies such as GWAS has led to the discovery of new biological mechanisms in TB. Transcriptomic approaches are powerful tools to derive host and pathogen molecular signatures and for identification of biomarkers for diagnosis and prognosis. However, both these approaches have their limitations that needs to be considered while designing future studies. There are inconsistencies in genomic studies due to the failure in replicating genetic associations across various studies and experimental settings; previous transcriptomic studies have highlighted the need to conduct comparisons across diverse disease cohorts and between whole blood, tissues, and cells, thus limiting the precise identification of genetic signatures between active and latent TB (90, 91). Proteomics, the complementary approach to genomics and transcriptomics which has been used to identify host-Mtb interactions and pathogen physiology, also suffers from limitations such as low instrument sensitivity (92). Although metabolomics provides the advanced omic platform for detection of small molecules, the precision of detection and measurement are affected by extraction method and conditions. There are variations in metabolomic analyses performed across research labs due to the variations in instrument, sample preparations, data integration and statistical analysis which currently limits the reproducibility, sensitivity and specificity required for clinical applications (93). The most recent fluxomic technology measures the metabolic phenotype of a biological system and provides a systems-wide identification of active pathways. However, there exists limitations in application of fluxomics to TB disease cohorts, firstly due to the unavailability of a human TB metabolic and mathematical model of disease and secondly, fluxomics is currently limited to measuring mainly central carbon fluxes in host and pathogen. Isotopic labelling studies in Mtb are mainly focussed on single isotope species. Co-labelling studies with different isotopes such as ^13^C and ^15^N are limited mainly because of the low sensitivities of MS instruments to precisely distinguish between different isotopic species. As evident from several studies, there are multiple nutrients that the TB pathogen utilises inside the human host. To gain a complete picture of the metabolic characteristics of Mtb pathogen in humans, there is a need to further develop the fluxomic technologies to model multiple substrate utilisation and carbon and nitrogen co-metabolic reactions. There is a need to develop metabolomics and MS sensitivities to precisely distinguish and measure isotopic species-specific MIDs obtained from labelling experiments conducted with multiple isotopic substrates. Finally, the flux computational platform needs to be developed to accurately model fluxes for the utilisation of multiple atoms through the metabolic network.

In conclusion, systems-based omic technologies have advanced the frontiers of host and pathogen biology and their interactions in TB disease. Over decades, there has been several successful attempts in developing and applying various omic platforms for the identification of diagnostic markers and therapeutic interventions. However, the current limitations in these omic approaches limit the investigation of host-pathogen molecular interactions in clinical settings and to reproducibly identify and validate diagnostic and therapeutic markers. The integration of various omic approaches, data sharing, cross lab validations and application to studying multiple disease cohorts are plausible ways forward to harness the calibre of various omic approaches for discovering clinically relevant biological mechanisms in TB.

## Author Contributions

KB, conceptualisation, writing, reviewing and editing, and funding acquisition. JM, reviewing and editing and funding acquisition. YX, writing and editing. All authors contributed to the article and approved the submitted version.

## Funding

This study was supported by the Biotechnology and Biological Sciences Research Council (BBSRC) grant, BB/V010611/1 United Kingdom. The funders have no role in preparation of this work.

## Conflict of Interest

The authors declare that the research was conducted in the absence of any commercial or financial relationships that could be construed as a potential conflict of interest.

## Publisher’s Note

All claims expressed in this article are solely those of the authors and do not necessarily represent those of their affiliated organizations, or those of the publisher, the editors and the reviewers. Any product that may be evaluated in this article, or claim that may be made by its manufacturer, is not guaranteed or endorsed by the publisher.
